# Institutional Notes and News

**Published:** 1910-03-12

**Authors:** 


### Institutional Notes and News.

**Middlesex Hospital and the L.C.C.**

The report for the year of the Middlesex Hospital was considered at the annual governor's court a few days ago. It states that the hospital after careful consideration has felt compelled to reject the L.C.C. scheme by which preferential treatment should be given in the out-patient department to children of the public elementary schools, on the ground that its acceptance means that the hospital's energies would then be influenced undesirably by pecuniary considerations. Both in-patients and out-patients increased in numbers during the year, the figures being, in-patients 5,470, out-patients 51,318, with an average of nearly three attendances each. The total income was £25,864, and the expenditure £36,951, which leaves a balance on the wrong side of £11,086. The income unfortunately has shown a decrease of £3,150, which the report attributes to the reduced receipts from legacies given specifically for the general purposes of the institution. This unfortunate decline in income has caused loans for current expenses to be contracted, and these represent a total indebtedness of £15,000, which will have to be increased shortly by £10,000 more. Prince Francis of Teck, who presided, said his presence there was due to his desire to appeal to the public, and to point out to it that the capital sum received by the hospital from the Barnato trustees is to be used to provide the income for the new extra wards which are being built in close relation to the cancer charity. The general fund of the hospital will gain nothing from that source.

**Westminster Hospital.**

In spite of legacies, the overdraft at the bank of Westminster Hospital is £2,800. The accounts themselves read as follows: Ordinary income £12,294, and expenditure £20,024, leaving a very serious discrepancy. The in-patients totalled 2,653, the out-patients 20,224, whose attendances, averaging almost four each, numbered 72,082. The court of Governors at which this report was presented held its annual meeting recently, and was presided over by Sir J. Wolfe Barry, a vice-president of the hospital.

**West Suffolk General Hospital.**

A busy year with many efforts in varied ways ended successfully is recorded in the annual report of the Bury and West Suffolk General Hospital. Almost exactly a year ago it was decided, because of the increasing work to be done, that the medical officers should be increased from three to four, the office of assistant medical officer being abolished and a further medical officer being appointed. That this was necessary is shown by the fact that the out-patient department has been practically rebuilt, and a new dispensary and additional rooms have been constructed; we may note that the cost of this work, some £540, has been subscribed for. Besides this, Earl Cadogan, the hospital's President, is providing the money for the rebuilding of the kitchens; and the balconies which were given by Mr. and Mrs. Charters, and which he opened, as The Hospital reported in October last, are in brisk use at present. Apart from this record of expansion, 624 in-patients have been treated (the stay of each averaging 26 days), and 2,547 patients have attended the new out-patient department, an increase of nearly 300 on those received in 1908. It is satisfactory, in view of this, to learn from Dr. Kilner that the work is going on smoothly and well. This eulogy is reflected in the balance sheet, which reads as follows: £3,357, received from all sources, and £3,362 expended all round. There have been two changes during the year in the staff appointments, Mr. E. Johnson having become house surgeon and Mr. H. E. Tracy dental surgeon to the hospital.

**Royal Victoria Hospital, Dover.**

The Royal Victoria Hospital, Dover, has had a satisfactory year, as was recorded at the annual meeting in February. In-patients increased to 501, and out-patients to 5,156, while the visits which the house surgeon paid to the homes of out-patients numbered 1,490. On the financial side of the report, it should be remembered, first, that in 1908 there was a deficit of £557, and a sub-committee was appointed to consider how this might be altered. Perhaps the best comment on the efforts of this committee is the fact that the deficit has now been reduced to £35. It must have required solid work to raise, as was done last year, the annual subscriptions by £150; for these are the most satisfactory source of income a hospital can have. The income amounted to £2,483, and the expenditure to the same sum, while £344 was received from legacies. The income for the Children's Ward now amounted to £202. Mr. Charles Wood, honorary acting medical officer, resigned after a long connection with the hospital, and Dr. Adamson was elected to succeed him. Sir W. Crundall was re-elected President of the hospital for the year.

**Sir E. Shackleton and the Seamen's Hospital.**

The Seamen's Hospital Society ("Dreadnought") was fortunate in securing Sir Ernest Shackleton to preside at the annual Court of Governors last Monday, and Prince's, where the meeting was held, had a distinguished and interested audience to listen to the details of the annual report. It was read by Mr. Percival Nairne (chairman of the Society), who emphasised the fact that the deficit was due to the falling-off in legacies, both annual subscriptions and donations having shown a considerable rise. The total number of patients treated during the year was 34,300. Sir Ernest Shackleton then pointed out that the shipping which brought the country's food to England was manned by men practically unknown to the people on shore. A sailor himself, he could understand what the Seamen's Hospital did for the men on these tramp steamers, men who were, as a class, almost beyond the reach of hospitals. On these grounds alone he urged that the Seamen's Society was worthy of support. Sir Ernest's election to an honorary life-governship of the hospital was then carried.

**Andover Cottage Hospital.**

The total number of patients admitted to the Andover Cottage Hospital during the year was 140, and that fact makes the opening statement of the report that this institution "has become almost a necessity to the town" a rather humorous and over modest one, especially as this number is 25 more than that of 1908. The total receipts were £517, total expenditure £385, with the highly satisfactory balance to the good of £132, or rather £77, as the Christmas bills, amounting to £55, were too late to be included in the financial accounts, which are closed on December 31. The salary of the matron, Miss Pütz, was raised by general agreement at the annual meeting to £50. The Free Bed Fund of the hospital, which has been showing a varying return for some years, last year realised an increase, owing to the special efforts that have been given to its furtherance.

